# Methods to measure effects of social accountability interventions in reproductive, maternal, newborn, child, and adolescent health programs: systematic review and critique

**DOI:** 10.1186/s41043-020-00220-z

**Published:** 2020-12-07

**Authors:** Cicely Marston, Catherine R. McGowan, Victoria Boydell, Petrus Steyn

**Affiliations:** 1 DEPTH Research Group, Department of Public Health, Environments & Society, Faculty of Public Health and Policy, London School of Hygiene & Tropical Medicine, 15-17 Tavistock Place, London, WC1H 9SH UK; 2 Global Health Centre, Geneva Graduate Institute, Chemin Eugène-Rigot 2, 1202 Genève, Switzerland; 3 UNDP/UNFPA/UNICEF/WHO/World Bank Special Programme of Research, Development and Research Training in Human Reproduction (HRP Research), World Health Organization, Geneva, Switzerland

**Keywords:** Social accountability, Methodology, Systematic review, Sexual health, Reproductive health, Maternal health, Newborn health, Child health, Adolescent health

## Abstract

**Background:**

There is no agreed way to measure the effects of social accountability interventions. Studies to examine whether and how social accountability and collective action processes contribute to better health and healthcare services are underway in different areas of health, and health effects are captured using a range of different research designs.

**Objectives:**

The objective of our review is to help inform evaluation efforts by identifying, summarizing, and critically appraising study designs used to assess and measure social accountability interventions' effects on health, including data collection methods and outcome measures. Specifically, we consider the designs used to assess social accountability interventions for reproductive, maternal, newborn, child, and adolescent health (RMNCAH).

**Data sources:**

Data were obtained from the Cochrane Library, EMBASE, MEDLINE, SCOPUS, and Social Policy & Practice databases.

**Eligibility criteria:**

We included papers published on or after 1 January 2009 that described an evaluation of the effects of a social accountability intervention on RMNCAH.

**Results:**

Twenty-two papers met our inclusion criteria. Methods for assessing or reporting health effects of social accountability interventions varied widely and included longitudinal, ethnographic, and experimental designs. Surprisingly, given the topic area, there were no studies that took an explicit systems-orientated approach. Data collection methods ranged from quantitative scorecard data through to in-depth interviews and observations. Analysis of how interventions achieved their effects relied on qualitative data, whereas quantitative data often raised rather than answered questions, and/or seemed likely to be poor quality. Few studies reported on negative effects or harms; studies did not always draw on any particular theoretical framework. None of the studies where there appeared to be financial dependencies between the evaluators and the intervention implementation teams reflected on whether or how these dependencies might have affected the evaluation. The interventions evaluated in the included studies fell into the following categories: aid chain partnership, social audit, community-based monitoring, community-linked maternal death review, community mobilization for improved health, community reporting hotline, evidence for action, report cards, scorecards, and strengthening health communities.

**Conclusions:**

A wide range of methods are currently being used to attempt to evaluate effects of social accountability interventions. The wider context of interventions including the historical or social context is important, as shown in the few studies to consider these dimensions. While many studies collect useful qualitative data that help illuminate how and whether interventions work, the data and analysis are often limited in scope with little attention to the wider context. Future studies taking into account broader sociopolitical dimensions are likely to help illuminate processes of accountability and inform questions of transferability of interventions. The review protocol was registered with PROSPERO (registration # CRD42018108252).

## Background

Accountability is increasingly seen as central to improving equitable access to health services [1, 2]. Despite the fact that social accountability mechanisms are “multiplying in the broader global context of the booming transparency and accountability field” [3, p. 346], whether and how these interventions work to improve health is often not adequately described. Measuring effects of social accountability interventions on health is difficult and there is no consensus on how social accountability should best be defined, developed, implemented, and measured.

The term accountability encompasses the processes by which government actors are responsible and answerable for the provision of high-quality and non-discriminatory goods and services (including regulation of private providers) and the enforcement of sanctions and remedies for failures to meet these obligations [4]. The Global Strategy for Women’s, Children’s and Adolescents’ Health, 2016–2030 defines accountability as one of nine key action areas to, “end preventable mortality and enable women, children and adolescents to enjoy good health while playing a full role in contributing to transformative change and sustainable development” [2 p. 39]. The Global Strategy’s enhanced Accountability Framework further aims to “establish a clear structure and system to strengthen accountability at the country, regional, and global levels and between different sectors” [2].

Social accountability, as a subset of accountability more broadly comprises “…citizens’ efforts at ongoing meaningful collective engagement with public institutions for accountability in the provision of public goods” [5 p. 161]. It has transformative potential for development and democracy [1, 6–9]. Successful efforts depend on effective citizen engagement, and the responsiveness of states and other duty bearers [3, 10]. Social accountability and collective action processes may contribute to better health and healthcare services by supporting, for example, better delivery of services (e.g., via citizen report cards, community monitoring of services, social audits, public expenditure tracking surveys, and community-based freedom of information strategies); better budget utilization (e.g., via public expenditure tracking surveys, complaint mechanisms, participatory budgeting, budget monitoring, budget advocacy, and aid transparency initiatives); improved governance outcomes (e.g., via community scorecards, freedom of information, World Bank Inspection Panels, and Extractives Industries Transparency Initiatives); and more effective community involvement and empowerment (e.g., via right to information campaigns/initiatives, and aid accountability mechanisms that emphasize accountability to beneficiaries) [10–12].

An early attempt to evaluate a social accountability intervention using an experimental study design was a 2009 paper presenting the evaluation of community-based monitoring of public primary health care providers in Uganda by Bjorkman and Svensson [13]. The authors conclude that, “…experimentation and evaluation of new tools to enhance accountability should be an integral part of the research agenda on improving the outcomes of social services” [13 p. 26]. Since then, various study designs have been used to assess social accountability initiatives. These include randomized trials, quantitative surveys, qualitative studies, participatory approaches, indices and rankings, and outcome mapping [10].

In common with other fields, social accountability interventions are increasingly popular in the area of reproductive, maternal, neonatal, child, and adolescent health (RMNCAH). Also in common with the broader area of social accountability, measuring effects of these interventions on RMCAH is challenging.

In this paper, we review and critically analyze methods used to evaluate the health outcomes of social accountability interventions in the area of RMNCAH, to inform evaluation designs for these types of interventions.

## Methods

### Eligibility criteria

We searched for original, empirical studies published in peer-reviewed journals between 1 January 2009 and 26 March 2019 in any language. We included papers which described an evaluation of the health effects of interventions aiming to increase social accountability of the healthcare system or specific parts of the healthcare system, within a clearly defined population. We included papers that reported one or more RMNCAH outcome. Because many papers did not include direct health outcome measures or commentary, we also included studies that reported on health service outcomes such as improvements in quality, on the grounds that this was likely to have some effect on health. Because we were interested in methods for measuring effects of social accountability interventions on health, we excluded papers that did not report at least one health (RMNCAH) outcome, for instance we excluded papers which only discussed how the intervention had been set up or how it was received and did not mention any health-related consequences of the interventions.

We excluded papers that described only top-down community health promotion type initiatives (e.g., improving community response to obesity); interventions aiming to improve accountability of communities themselves (e.g., community responsibilities toward women during childbirth); clinician training interventions (e.g., to reduce abuse of women during childbirth); quality improvement interventions for clinical care (e.g., patient participation in service quality improvement relating to their own care and treatment and not addressing collective accountability); intervention development (e.g., testing out report cards as there was no evaluation of the effects of using these); natural settings where people held others to account (i.e., there was no specific intervention designed to catalyze this); or papers that exclusively discussed litigation and legal redress.

### Information sources

 We searched the following databases via Ovid: MEDLINE, EMBASE, and Social Policy & Practice. Both SCOPUS and The Cochrane Library were searched using their native search engines. All database searches were carried out on 28 August 2018 and updated on 26 March 2019. We reviewed reference  lists and consulted subject experts to identify additional relevant papers.

### Search

We developed search terms based, in part, on specific methods for achieving social accountability as defined in Gaventa and McGee 2013 [10]. The search combined three domains relating to accountability, RMNCAH, and health. The complete search strategy used for all five databases is included in Table 1.

**Table 1:** Search terms

Accountability^a^	**Social Responsibility [MeSH]**^b^**, Community Participation [MeSH]**, accountab^a^, “collective action”, “community action”, social mobili#ation”, “community mobili#ation”, “social movement^a^”, “community movement^a^”, “participatory budgeting”, “public expenditure tracking”, “citizen charter^a^”, “public hearing^a^”, “citizen report card^a^”, “social audit^a^”, “health committee^a^”, “community scorecards”, “complaint mechanism^a^”, “social protest^a^” [TiAb]
And	
Reproductive health	**Reproductive Health [MeSH], Sexual Health [MeSH], Contraception [MeSH], Sexually Transmitted Diseases [MeSH]**, HIV, STI, “sexually transmitted infection^a^”, reproductive, “sexual health”, “family planning”, contracept^a^, abortion^a^ [TiAb]
Maternal health	**Maternal Health [MeSH]**, maternal [TiAb]
Newborn health	**Infant Health [MeSH]**, infant^a^, newborn^a^ [TiAb]
Child health	**Child Health [MeSH]**, child^a^ [TiAb]
Adolescent health	**Adolescent Health [MeSH]**, adolescen^a^, youth, young people, young person [TiAb]
AND	
Health	**Health [MeSH]**, health^a^, wellness [TiAb]

^a^The Cochrane Library was searched using only the accountability terms

^b^MeSH terms are specific to MEDLINE. The searches of EMBASE, Social Policy & Practice, and SCOPUS were as above but without MeSH terms

### Study selection

Papers were screened on title and abstract by CM and CRM and lack of agreement was resolved by VB. Full text papers were screened by CM and VB.

### Data collection and data items

Data were extracted by CM and CRM. Data items included intervention, study aims, population, study design, data collection methods, outcome measures, social accountability evidence reported/claimed, cost, relationship between evaluator and intervention/funder, which theoretical framework (if any) was used to inform the evaluation, and if so, whether or not the evaluation reported against the framework.

Social interventions are complex and can have unexpected consequences. Because these may not always be positive, we were interested to explore how this issue had been addressed in the included studies. We extracted from the studies any discussion of how such negative effects were measured, whether they were measured, and whether any such effects were reported on. We defined harms and negative effects very broadly and included any consideration at all of negative impacts or harms, even if they were mentioned only in passing.

Because we were examining accounts of interventions that increase accountability in various ways, we were interested in the extent to which the authors included information that would promote their own accountability to their readers. We examined whether the studies contained information about the funding source for the intervention and for the evaluation, or any other information about possible conflicts of interest.

### Risk of bias

For this review, we wished to describe the study designs used to evaluate social accountability interventions to improve RMNCAH. Papers reporting on interventions that aimed to affect comprehensive health services where the studies did not explicitly reference RMNCAH components (or which have not been indexed in MEDLINE using related keywords and/or MeSH terms) were not included. Interventions in general areas of health are likely to employ similar methods to evaluate social accountability interventions as those in RMNCAH-specific areas. However, if not, these additional methods would not have appeared in our search and will be omitted from the discussion below.

### Synthesis of results

We present a critical, configurative review (i.e., the synthesis involves organizing data from included studies) [14] of the methodologies used in the included evaluations. We extracted data describing the social accountability intervention and the evaluation of it (i.e., evaluation aims, population, theoretical framework/theory of change, data collection methods, outcome measures, harms reported, social accountability evidence reported, cost/sustainability, and relationship between the funder of the intervention and the evaluation team). We presented the findings from this review at the WHO Community of Practice on Social Accountability meeting in November 2018, and updated the search afterwards to include more recent studies.

### Registration

The review protocol is registered in the PROSPERO prospective register of systematic reviews (registration # CRD42018108252).^1^ This review is reported against PRISMA guidelines [15].

## Results

**Fig. 1:**
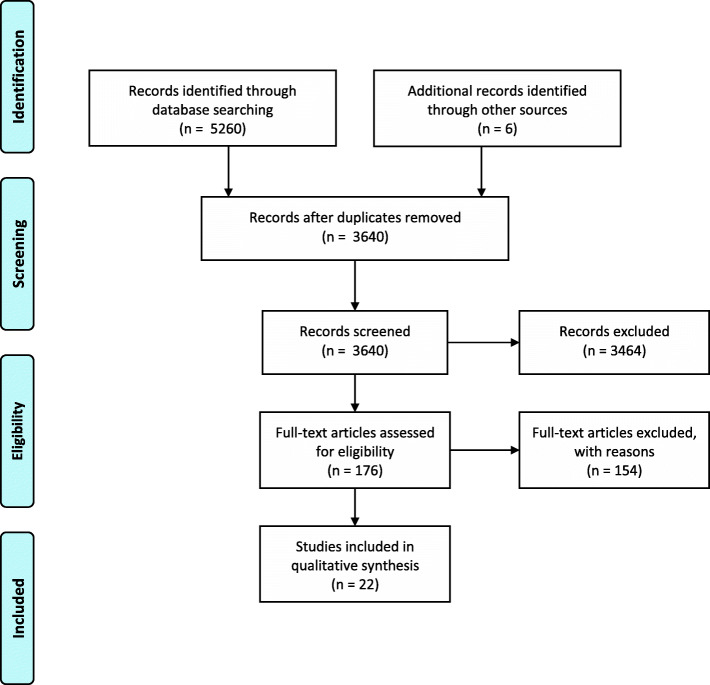
PRISMA 2009 flow diagram

The search yielded 5266 papers and we found an additional six papers through other sources. One hundred and seventy-six full text papers were assessed for eligibility and of these, 22 met the inclusion criteria (Fig. 1).

### Interventions measured

We took an inclusive approach to what we considered to be relevant interventions, as reflected in our search terms. Our final included papers referred to a range of social accountability interventions for improving RMNCAH. Eight types of interventions were examined in the included papers (Table 2).

**Table 2:** Intervention types

Social accountability approach	Intervention type (i.e., social accountability mechanism)	Definition	Context
Participatory policy and budget analysis	Aid chain partnership	Aid chain partnerships are partnerships between international, governmental and civil society organisations to determine the distribution of international aid [1].	Cambodia [1]
Participatory public expenditure/input tracking	Social audit	A social audit process engages both service providers and communities to assess performance of health facilities against national service delivery standards [2].	Zambia [2]
Participatory healthcare service performance monitoring, evaluation, and quality improvement	Community-based monitoring	This aims to improve public services by encouraging people to document the availability, accessibility, and quality of public services against specific commitments or standards.	India [3, 4], Peru [5], Uganda [6], Zambia [7]
	Community-linked maternal death review	This was described as, “an alternative community-linked maternal death review (MDR) system combining the strengths of facility-based MDR and community verbal autopsy”. [8, p. 2]	Malawi [8]
	Community mobilisation for improved health, equity and rights.	Community mobilisation can be defined as, “…community members taking collective action to achieve a common goal related to health, equity and rights”. [9, p. 60]	India [10, 11], South Africa [9], United States [12]
	Community reporting hotline	These were free telephone hotlines for reporting poor service provision. This was implemented in India to enable women to report demands for informal payments [13].	India [13]
	Evidence for action	The E4A programme supported multiple interventions in six countries (Ethiopia, Ghana, Malawi, Nigeria, Sierra Leone, and Tanzania) including scorecards, dashboards, and maternal death reviews (MDRs).	Multi-site (Ethiopia, Ghana, Malawi, Nigeria, Sierra Leone, and Tanzania) [14, 15]
	Report cards	Data are collected from community members, often through a household survey, to rate a local health facilities performance against existing or pre-determined indicators and made available to communities in facilitated sessions using citizen report cards	Tanzania [16], Uganda [16, 17]
	Scorecards	Community members collectively identity and prioritize their concerns and barriers with local health services and then work with local health providers to jointly develop actions to address and monitor the issues. They differ from report cards in that the community determines what the priorities should be whereas report cards report against existing standards.	Ghana [18], Malawi [19–21], Zambia [2]
	Strengthening health communities (multi-method)	Strengthening health communities involves multiple accountability mechanisms aimed at strengthening the influence and representation of communities in improving their health systems.	India [11], Tanzania [22]

### Study aims

To be included in this review, all studies had to report on health effects of the interventions and be explicitly orientated around improving social accountability. The different studies had somewhat different aims, with some more exploratory and implementation-focused, and some more effectiveness-orientated. Exploratory studies were conducted for maternal death reviews [16], social accountability interventions for family planning and reproductive health [17], civil society action around maternal mortality [18], community mobilization of sex workers [19], community participation for improved health service accountability in resource-poor settings [20], and exploring a community voice and action intervention within the health sector [21]. These aimed to describe contextual factors affecting the intervention, often focusing more on implementation than outcomes. Others explicitly aimed to examine how the interventions could affect specific outcomes. This was the case for studies of an HIV/AIDS programme for military families [22]; effects of community-based monitoring on service delivery [13]; effectiveness of engaging various stakeholders to improve maternal and newborn health services [23]; acceptability and effectiveness of a telephone hotline to monitor demands for informal payments [24]; effectiveness of CARE’s community score cards in improving reproductive health outcomes [25]; assess effects of quality management intervention on the uptake of services [26]; examine structural change in the Connect 2 Protect partnership [27]; improve “intercultural maternal health care” [28]; and whether and how scale up of HIV services influenced accountability and hence service quality [29]. Some studies were unclear in the write up what the original aims were, but appeared to try to document both implementation and effectiveness, for example the papers reporting on scorecards used in Evidence4Action (E4A) [30, 31].

### Study designs used

Study designs varied from quantitative surveys to ethnographic approaches and included either quantitative or qualitative data collection and analysis or a mix of both (see Table 3). Direct evidence that the intervention had affected social accountability was almost always qualitative, with quantitative data from the intervention itself used to show changes, e.g., in health facility scores. The possibility that those conducting the intervention may have had an interest in showing an improvement which might have biased the scoring was not discussed.

**Table 3:** Evaluation study design and data collection methods of the included studies

Study	Social accountability intervention	Evaluation aims	Evaluation design/data collection
Aveling, E. L. (2010). “The impact of aid chains: Relations of dependence or supportive partnerships for community-led responses to HIV/AIDS?” AIDS Care Psychological and Socio-Medical Aspects of AIDS/HIV 22(SUPPL. 2): 1588–1597.	The Happy Couples Programme (HCP). “The HCP brings into ‘partnership’ international and local development organisations, government institutions and military families. The programme is an HIV/AIDS prevention programme using peer education with Cambodian soldiers and their wives, which aims to increase reproductive health knowledge, condom use and access to health services”. (p. 1589)	The evaluation, “…examines how aid relationships can both promote and undermine the possibility of successful community-led responses” to HIV/AIDS. (p. 1589)	Case study ethnography. Data collection methods included semi-structured, qualitative interviews review of documentary evidence, observations of programme activities.
Bayley, O., H. Chapota, E. Kainja, T. Phiri, C. Gondwe, C. King, B. Nambiar, C. Mwansambo, P. Kazembe, A. Costello, M. Rosato and T. Colbourn (2015). “Community-linked maternal death review (CLMDR) to measure and prevent maternal mortality: a pilot study in rural Malawi.” BMJ Open 5(4): e007753.	“The process was triggered in the event of any maternal death, by community CLMDR team members hearing about a death in their area. Stage 1 began with the woman’s family giving consent for the process, followed by a verbal autopsy, or structured interview, including multiple open-ended free-text questions about the events leading up to her death. This form was used to record data at all stages of the process and designed to facilitate discussion and communication between participants. Stage 2 was a meeting held in the woman’s local area by the community team. They recorded factors they believed contributed to the woman’s death, and suggested strategies to prevent future deaths. Stage 3 was a meeting held at the woman’s local health facility or at the district hospital dependent on where the death occurred, with a broad spectrum of health center staff, district hospital staff and the HSA. The HSA reported the information from the verbal autopsy and the community team discussions. Participants agreed on a medical cause of death and health facility factors that may have contributed to the death, after which they recorded the strategies that they planned to prevent future deaths. Action points were assigned to individual health center and district hospital staff to implement. Stage 4 was a public meeting held in the woman’s local community, attended by district hospital and health center representatives, the HSA, community leaders, and community members—all were welcome to attend. The HSA sought the family’s consent to summarize the case in order to facilitate an open discussion of all relevant factors. The health workers presented their planned action points. The community agreed on community factors that may have contributed to the death and planned their own strategies, assigning action points for individuals to implement. Stage 5 was a bimonthly meeting, which provided an opportunity for community and health facility representatives to hear about progress on implementing action points, celebrate successes, and to identify and overcome any barriers to action. An additional meeting of traditional leaders was held quarterly in order to share innovations and lessons learned across the whole district.” (p. 3–4)	"Our study describes the Malawian context and identifies six weaknesses of the current MDR system. We present the pilot study of the CLMDR process over a 1-year period, and the results of how it can overcome these weaknesses and provide an estimate of maternal mortality. We conclude with thoughts on the added value and applicability of the CLMDR approach". (p. 2)	No specific evaluation design provided. The paper discusses the process of conducting death reviews in the community, i.e., the design of the intervention rather than the evaluation.
Björkman M, Svensson J. Power to the People: Evidence from a Randomized Field Experiment on Community-Based Monitoring in Uganda. The Quarterly Journal of Economics. 2009;124(2):735-69.	Community mobilization through participatory surveys that solicit user feedback on the performance of public services against set standards “…aimed at enhancing community involvement and monitoring in the delivery of primary health care…” (p. 739)	“To examine whether community-based monitoring works, we designed and conducted a randomized field experiment in fifty communities from nine districts in Uganda”. (p. 736) "First, data were required to assess how the community at large views the quality and efficacy of service delivery. We also wanted to contrast the citizens’ view with that of the health workers. Second, data were required to evaluate impact." (p. 740)	“Two surveys were implemented: a survey of the fifty providers and a survey of users. Both surveys were implemented prior to the intervention (data from these surveys formed the basis for the intervention) and one year after the project had been initiated” (p. 740–741)
Blake, C., N. A. Annorbah-Sarpei, C. Bailey, Y. Ismaila, S. Deganus, S. Bosomprah, F. Galli and S. Clark (2016). “Scorecards and social accountability for improved maternal and newborn health services: A pilot in the Ashanti and Volta regions of Ghana.” International Journal of Gynecology and Obstetrics 135(3): 372–379.	“The initiative was designed to strengthen partnerships between clients, providers, and the community at large for improved maternal and newborn health (MNH) care through a social accountability process using scorecards. Before carrying out scorecard assessments, health providers and community-based NGOs were trained on MNH rights and client care to ensure a common understanding of entitlements in MNH service delivery. Although this intervention did not focus on clinical skills building for quality EmONC, the aim was to improve the enabling environment for EmONC and engage the community at large in this endeavor.” (p. 373)	The aims were to, “…examine qualitative and quantitative evidence from the social accountability intervention used by Evidence 4 Action to assess the effectiveness of engaging multiple health and non-health sector stakeholders to improve MNH services at facility level. It also identifies some limitations to this strategy and makes recommendations for future interventions of a similar nature.” (p.373)	“The study had two components. The quantitative component comprised two rounds of facility assessments. The qualitative component prospectively assessed the impact of changes in policy, attitudes, and/ or practices.” (p. 373) “An independent prospective policy study carried out by external researchers followed the E4A program with the aim of understanding the resulting changes at district and regional level. Data collection focused on process tracing to assess whether and how the scorecard process contributed to changes in policies or to changes in attitudes or practices among key stakeholders. This data collection included regular meeting observations and analysis of documents, as well as repeat interviews with a broad base of MNH actors, including local government staff, district assembly members, health facility managers, community leaders, and organizations.” (p. 374) [see Nove et al for more information about Evidence 4 Action]
Boydell, V., S. Neema, K. Wright and K. Hardee (2018). “Closing the Gap between People and Programs: Lessons from Implementation of Social Accountability for Family Planning and Reproductive Health in Uganda.” African Journal of Reproductive Health 22(1): 73–84.	“Between 2008 and 2013, the German Foundation for World Population (DSW) and Reproductive Health Uganda (RHU) implemented the European Commission-funded HAP in five districts in Uganda. HAP was a social accountability project aimed at empowering civil society and citizens in Uganda to effectively participate in policy priorities, monitoring their implementation and holding duty bearers to account for their promises. The project outlined a theory of change (TOC) which informed the project‘s actions. First the capacity of civil society organizations (CSOs) for advocacy, resource mobilization and civic education was built. With this improved capacity, the CSOs then could mobilize communities to participate in monitoring services and actively interact with decision-makers to generate solutions and work in partnership to bring about the desired changes. The project started with selecting national CSOs for a series of capacity building activities. The trained CSOs received a sub-award to support community activities such as (1) increasing community members‘ awareness about family planning and their entitlements; (2) building civil society and community coalitions at the district level; (3) undertaking civic education with local communities; and (4) training community members on holding dialogues with health care providers and health officials to jointly identify challenges, priorities and solutions.” (p. 75)	The paper assessed "results of retrospective implementation research into a five-year social accountability project in Uganda that focused on family planning and reproductive health. A mix of methods was used examine the project‘s implementation in three districts in Uganda between 2009 to 2013” (p.73) “To address these gaps this paper presents findings from an implementation study of a social accountability project focused on FP and reproductive health (RH), the Healthy Action Project (HAP), implemented in Uganda between 2009 and 2013. This paper examines the implementation of HAP with a focus on family planning.” (p. 74)	Retrospective implementation research using document review, Political Economy Analysis, and interviews.
Dasgupta, J. (2011). “Ten years of negotiating rights around maternal health in Uttar Pradesh, India.” BMC International Health and Human Rights 11 (SUPPL. 3) (S4).	“This paper reviews documents of the last ten years describing the experiences of a Non-Governmental Organization, SAHAYOG, in working with a civil society platform, the Healthwatch Forum, to develop ‘rights based’ strategies around maternal health. The paper builds an analysis using recent frameworks on accountability and gendered rights claiming to examine these experiences and draw out lessons regarding rights claiming strategies for poor women.” (from abstract)	“This paper interrogates the process of civil society action around maternal mortality in Uttar Pradesh to ask why the issue of maternal deaths never becomes a ‘political’ issue, why the agent of accountability is never clear and despite some gains at the localized sites, overall why the health system and bureaucracy remain inert; and what needs to be done differently.” (p.4)	Reflective, qualitative study. The paper uses “organizational records, including unpublished internal and external evaluation reports, in-house publications and web-based documents describing the experiences of SAHAYOG’s work of the last ten years […and…] unpublished reports of community based participatory approaches […], and several rounds of ‘policy dialogues’.” (p.4) “Armed with information about their entitlements and state provisions, the MSAM women, in an exercise of ‘active citizenship’ through monitoring and advocacy, took up various aspects of the NRHM each year for interrogation. At the start they revealed local corruption in the appointment of the ASHA workers (2006), then they audited the payment of the conditional cash transfer under the JSY (2007-8); they examined how ‘untied’ health budgets are spent locally and how much poor families are spending (2009), and recently they audited the compliance of health sub-centres with the Indian Public Health Standards (2010). Every NRHM monitoring exercise was followed by a formal presentation to the district health officials by the NGOs and MSAM women, as well as presentations at the state capital, Lucknow, usually in the presence of state officials and the media.” (p. 7)
Dasgupta, J., Y. K. Sandhya, S. Lobis, P. Verma and M. Schaaf (2015). “Using Technology to Claim Rights to Free Maternal Health Care: Lessons about Impact from the My Health, My Voice Pilot Project in India.” Health Hum Rights 17(2): 135–147.	“The Mera Swasthya, Meri Aawaz pilot project was developed to test whether a free telephone hotline connected to Ushahidi (www.ushahidi.com)—an open-source data management system that aggregates and displays data—could be tailored for illiterate women and used to monitor demands for informal payments. The implementers also sought to understand how the project could inform and strengthen grassroots advocacy efforts around maternal health, how it could affect women’s ability to claim their rights to maternal health care, and whether scale-up was feasible. To that end, it documented factors that contributed to success and failure, the project’s adaptation over time, challenges, and remaining questions.” (p.138) “The system works as follows: Women call the toll-free hotline to report having been asked to pay informal payments at a hospital. Each hospital in the project’s districts is assigned a four-digit code. Callers are asked to enter the hospital’s four-digit code as well as additional codes corresponding to the amount and purported justification for the payment (for example, “Press 2 if money was requested to pay for drugs.”) The information collected is then mapped in an Ushahidi installation and can be viewed at www.meraswasthyameriaawaz.org. Callers reporting emergencies are immediately routed to a live person; the emergency line is staffed 24 hours a day by a representative of the partnering community-based organizations.” (p. 139)	To assess the acceptability and effectiveness of the pilot project.	Qualitative mixed methods plus quantitative records of reports to the hotline (causality not directly assessed, a limitation the authors acknowledge) “By the end of the project, MSAM members were known for challenging informal payments. Therefore, some primary health clinic staff stopped demanding informal payments once they knew that the woman was in some way affiliated with MSAM. The staff tended to treat women better in such cases” (p.142) “One example comes from Azamgarh. Through focus group discussions with MSAM women, we learned that following a block-level sharing of the Mera Swasthya, Meri Aawaz data, government officials took immediate action to remedy problems identified at one facility, including by fixing the water supply, improving electricity, providing free medicines, and offering food to women in the hospital following delivery. In addition, staff behavior toward women improved. The additional director of the Azamgarh District stated that the act of registering complaints was very important and that the Ushahidi data was useful because it made officials realize the enormity of the problem. Our analysis of the reporting patterns showed that the number of reports made about this particular facility dropped from an average of 18 reports per month before the block-level dialogue (January to November 2012) to 3 reports per month after the dialogue. The comments of the additional director and others lead us to believe that this decrease in reports was likely because requests for informal payments decreased. In this case, the dialogue was a catalytic event, as it triggered positive changes that included not only reductions in demands for informal payments but also improvements in staff behavior and infrastructure. The success of this dialogue also provided a boost to the MSAM women’s confidence in their ability to effect improvements in their health facilities.” (p. 143)
de Souza, R. (2009). “Creating ‘communicative spaces’: a case of NGO community organizing for HIV/AIDS prevention.” Health Communication 24(8): 692–702.	“The goal of the project is to reduce the prevalence of HIV/AIDS among female sex workers through participatory and empowering process of information dissemination and capacity building…The project will enable the sex worker community to participate in planning, monitoring, and evaluating interventions that affect their lives” (Project proposal, quoted in methods section, p. 694)	“What are the processes NGOs use to organize marginalized communities for HIV/AIDS prevention?” (p. 694) The study “seeks to understand ways in which the ideal of civil society can be achieved through more “civil” practices […] a case wherein an NGO uses practices within the discursive sphere to resurrect community voices.” (p. 694)	This research on an existing programme used a case study approach; “the case study method allows for data to be collected in a variety of ways (e.g., interviews, focus groups, and documents), thereby allowing for these complex processes to be identified. Finally, the case study method was used because, as with other ethnographic methods, it allows for attention to be given to the sociocultural context within which behaviors occur, an important feature of the culture-centered approach.” (p. 694)
George, A. S., D. Mohan, J. Gupta, A. E. LeFevre, S. Balakrishnan, R. Ved and R. Khanna (2018). “Can community action improve equity for maternal health and how does it do so? Research findings from Gujarat, India.” Int J Equity Health 17(1): 125.	Raising awareness, community monitoring, and dialogue with government health providers and authorities including use of report cards. (p. 9)	The study assesses the effects of community action on access to facility deliveries by marginalized groups across public and private sectors. It also evaluates the implementation processes that underpin community action and accountability for maternal health in Gujarat, India. (p. 2)	Document review, interviews, non-probability sample questionnaire survey. “The study combined qualitative data (project documents and 56 stakeholder interviews thematically analyzed) with quantitative data (2395 women's self-reported receipt of information on entitlements and use of services over 3 years of implementation monitored prospectively through household visits). Multivariable logistic regression examined delivery care seeking and equity.” (from Abstract). “The data are not based on any pre-determined sampling design and represent the efforts of volunteers to gather data from as many women as possible” (p. 4)
Gullo S, Galavotti C, Kuhlmann AS, Msiska T, Hastings P, Marti CN. Effects of a social accountability approach, CARE's Community Score Card, on reproductive health-related outcomes in Malawi: A cluster-randomized controlled evaluation. PLoS ONE. 2017;12 (2), e0171316.	Scorecards (see below)	“To this end, we designed a cluster-randomized control evaluation to assess the effectiveness of CARE’s Community Score Card (CSC) [11], a social accountability approach, to improve reproductive health-related outcomes in Ntcheu, Malawi”. (p. 2)	“CARE’s CSC was assessed in a cluster-randomized trial in the catchment areas of20 health facilities the in Ntcheu district of Malawi.” (p. 3) See Gullo et al. 2018 for further details.
Gullo, S., A. S. Kuhlmann, C. Galavotti, T. Msiska, C. Nathan Marti and P. Hastings (2018). “Creating spaces for dialogue: a cluster-randomized evaluation of CARE's Community Score Card on health governance outcomes.” BMC Health Services Research 18(1): 858.	“The CSC [community score card] intervention consists of five phases… planning and preparation… involves identifying the sectoral and geographic scope of the initiative, understanding the context and barriers both service providers and users face, training facilitators, and securing cooperation and buy-in from all participating parties, including government officials. In phase 2, the CSC is conducted with the community via focus group discussions with community members (separated into groups such as men, women, youth, etc.) to identify and prioritize issues they are facing in accessing services. Identified issues are organized into themes and a measureable indicator is developed for each theme. The indicators are then verified and scored by the community, generating a Score Card. The community also indicates reasons for why a particular score was given and creates suggestions for improvement. The same process of issue generation and indicator development is conducted with service providers in phase 3; through focus group discussions, service providers identify issues they are facing in delivering quality services, develop and score indicators, give reasons for the scores, and make suggestions for improvement. Phase 3 can occur either after or concurrently with phase 2. The CSC comes to life in phase 4 at the interface meeting, during which community members and service providers are joined by local government officials and other power holders to share and discuss their respective Score Cards, issues and priorities. This joint conversation gives way to locally identified solutions and a community-wide action plan for service improvement. Finally, phase 5 involves action plan implementation, monitoring, and evaluation in which community members, service providers, government staff and additional power-holders all have a role to play in reviewing and monitoring progress on indicators. This cycle is repeated (minus the initial planning and preparation stage) every six months: communities and service providers reconvene to discuss issues (and generate new ones, if needed), re-score the indicators and discuss reasons for changes, and then meet in an interface meeting to review their respective Score Cards, in an on-going cycle of problem identification, solution generation, implementation of improvements, and mutual accountability.” (p. 4)	Evaluate programme: “CARE’s Community Score Card (CSC), a social accountability approach, to improve reproductive health-related outcomes in Ntcheu, Malawi. The theory of change underlying the CSC intervention suggests that bringing together community members, health workers, and local officials to a) identify barriers and facilitators of service use and delivery, b) prioritize actions, and c) jointly monitor improvements will result in new and expanded spaces for inclusive, effective dialogue and negotiation. This, in turn, will empower both women and health workers in the community, leading to improved health behaviors, increased service utilization, and higher quality and more equitable service delivery. Ultimately, these changes, along with system and institutional changes, should decrease maternal and neonatal mortality in communities. Therefore, this evaluation aims to test the effectiveness of CARE’s CSC on maternal and reproductive health-related outcomes.” (p. 2)	“CARE’s CSC was assessed in a cluster-randomized trial in the catchment areas of 20 health facilities the in Ntcheu district of Malawi. Health facilities were matched in pairs and one facility from each pair was randomly assigned to participate in the CSC and the other was assigned as a control health facility. Two-stage cluster sampling was used to select group villages and villages for participation in the study.” (p.3) “We used data obtained from government census, district, and local office sources to construct the population from which we would draw the sample. Among intervention health facilities, there were 56 group villages (GVs) that contained 290 villages with a total population of 228,029. Among control health facilities, there were 36 GVs that contained 228 villages with a total population of 170,201. Using UNICEF’s probability-proportional-to-size (PPS) sampling method, we selected twenty GVs (i.e., clusters) from the intervention area and twenty GVs in the control area to serve as the primary sampling units. One of the largest intervention health facilities contained eight sampled GVs. Because we could not feasibly implement the CSC for all eight GVs in a single health facility, four GVs were dropped (leaving 16 GVs in the sample), and the PPS sample for this health facility was obtained from the remaining four GVs. The CARE Malawi team purposively identified 64 villages from the 16 intervention GVs in which to work, and randomly selected 64 villages in the 20 control GVs; the same PPS method described above was used to select villages. The number of individuals sampled in each village was determined by number of eligible women in a village multiplied by the sampling proportion for the condition (i.e., the required sample size divided by the total eligible population). We sized the sample to detect a 10% change in institutional births, based on the prevailing rates of institutional births in Ntcheu (78%), prior to baseline. Given the hypothesized effect size, our power analysis determined a sample of 650 women per treatment condition (power = .80, 2-tailed α = .05, non-response = 5%, and design effect = 2.0).” (p. 6) “Community members and service providers developed 12 indicators to track progress, for example, reception of clients at the facility, level of male involvement in maternal newborn health (MNH) issues, and availability of transportation for referrals during labor and delivery. CSC participants and service providers generated similar issues, but from their different perspectives. For example, ‘relationship with providers’ was an indicator for both: from the community side this referred to how providers treated them, whereas from the provider’s side, it referred to things like patients not listening to them, or following their guidance. The service providers also generated one additional indicator—availability of supervisory support—for a total of 13 Score Card indicators. In an open discussion, participants agreed on scores for each indicator using a scale from 0–100. This was done with the communities and the service providers separately, and then, during the interface meeting the Score Cards were discussed and actions to improve scores were agreed upon. For each intervention site, there were 1–4 community Score Cards and 1 service provider Score Card. The same indicators were used across all 10 intervention sites and were re-scored during each 6 month CSC cycle.” (pp.8–9)
Hamal, M., T. de Cock Buning, V. De Brouwere, A. Bardaji and M. Dieleman (2018). “How does social accountability contribute to better maternal health outcomes? A qualitative study on perceived changes with government and civil society actors in Gujarat, India.” BMC Health Serv Res 18(1): 653.	“The Government of Gujarat has taken several initiatives to improve maternal health services, such as the Chiranjeevi Yojana (free childbirth services for poor women in private health facilities), the Kasturba Poshan Sahay Yojana (financial assistance for poor pregnant women).” (p. 2)	“This study explores the existing social accountability mechanisms for maternal health, the factors they address and how the results of these mechanisms are perceived.” (From abstract)	Qualitative research using in depth interviews and focus group discussions with individuals from civil society and the government health system (*N* = 10, and with clients/beneficiaries (*N* = 26)). (p. 4)
Hanson C, Waiswa P, Marchant T, Marx M, Manzi F, Mbaruku G, et al. Expanded Quality Management Using Information Power (EQUIP): protocol for a quasi-experimental study to improve maternal and newborn health in Tanzania and Uganda (2014). Implementation Science 9(1):41.	Quality management: “QM involves applying a set of principles to improve quality: conceptualizing work as processes (e.g., following a case-management guideline), designing processes to reduce errors, focusing improvement efforts on the most vital processes, satisfying both clients and employees, monitoring quality, using scientific and statistical thinking, creating new organizational structures (e.g., quality improvement teams), and involving all workers in quality improvement. QM also includes a structured problem-solving methodology, which uses teams to improve quality with continuous plan-do-study-act (PDSA) cycles, which monitor indicators, identify problems, understand causes, implement solutions, check if solutions are working, and modify solutions as needed.” (p. 2)	“EQUIP aims to test whether a Quality Management (QM) approach at three levels of care and supported by district level report cards generated by continuous surveys can improve the quality and utilization of services for mothers and newborns”. (p. 8) “The specific objectives are: 1. To assess the effects of the EQUIP intervention on uptake and quality of care of key maternal and newborn health interventions; 2. To assess the feasibility and acceptability of the intervention; 3. To model the potential impact of the intervention using the Lives Saved Tool (LiST); 4. To estimate cost and cost-effectiveness of the intervention.” (p. 3)	[NB this is a protocol] “The evaluation compares intervention and comparison districts with respect to change in utilization and quality of healthcare using indicators of coverage, service quality and knowledge”. (p. 7) “During the entire study period, ongoing data collection via continuous, high quality household and heath facility surveys is used to estimate pre- and post-intervention outcome levels in one intervention and one non-randomly selected comparison district each in Uganda and Tanzania. The continuous household surveys and health facility censuses cover implementation and comparison districts. The QM intervention, supported by report cards using data generated by the continuous surveys, is implemented in intervention districts only. For evaluation, changes over time in quality and uptake of key maternal and newborn interventions in intervention areas are compared with changes over time in comparison areas, with careful attention paid to contextual factors that also vary over time” (p. 3) “A qualitative sub-study on feasibility and acceptability includes: how, when, and with what intensity the intervention is implemented in the intervention district; how the intervention worked at different levels; and changes and observations reported by QITs. In-depth interviews with district staff involved in the project are used to assess the acceptability of the QM approach and feasibility of implementation within the district structure. The evaluation uses a non-interrupted time-series approach to compare changes over time in primary outcomes (see below) in intervention and comparison areas. We generate a single estimate of effect for each primary outcome, adjusting for confounding factors and baseline levels. Provided that utilization, quality or coverage improves sufficiently for an effect on survival to be plausible, we will also use the Lives Saved Tool (LiST) to model the potential impact of the intervention on child survival” (p. 7)
Hulton, L., Z. Matthews, A. Martin-Hilber, R. Adanu, C. Ferla, A. Getachew, C. Makwenda, B. Segun and M. Yilla (2014). “Using evidence to drive action: A ‘revolution in accountability’ to implement quality care for better maternal and newborn health in Africa.’ Int J Gynaecol Obstet 127(1): 96-101.	"In 2011, the Commission on Information and Accountability (CoIA) for Women’s and Children’s Health published a report with 10 key recommendations […] Better use of information to improve results, better tracking of resources, and better oversight of results and resources globally and nationally were the three areas within which the CoIA recommended action. Making countries and stakeholders accountable for women’s and children’s health was a central tenet of the recommended actions. […] Evidence for Action (E4A)—a DFID-funded program that contributes to accountability in MNH—was launched soon after the publication of the Commission’s Final Report and was designed explicitly as a contribution to delivering the CoIA framework in six African countries: Ethiopia, Ghana, Malawi, Nigeria, Sierra Leone, and Tanzania. […] the program seeks to address resource deficiencies in these countries that contribute to suboptimal quality of MNH care. A strategic combination of evidence and advocacy to stimulate and strengthen accountability underpins this program.” (p. 96) “The E4A program therefore invests in catalytic interventions that stimulate accountability and involve the use of well-communicated evidence: first via better use of evidence by decision-makers and second via better communicated information as a basis for influencing public perceptions and political action.” (p. 97) “Data capture through scorecards, dashboards, and maternal death reviews (MDRs) are some of the ways in which E4A has been supporting evidence building and decision-making. For example, marshalling existing data into district dashboards (a visual display of key MNH indicators and other health resources consolidated on a single screen in the form of tables and graphs) that are communicated to and increasingly owned by district decision-makers has exposed the variation across districts and among facilities in Malawi, and stimulated discussions among them to make informed decisions about planning and resource allocation locally.” (p. 97)	“We aim to address the specific need for more “country-level detail about how the Commission’s recommendations are leading to change and action” as identified in the recent iERG report” (p. 97). “In addition to forming the basis for program design, E4A’s theory of change also provides us the scope to test whether data and evidence drive improvements through advocacy and social accountability.” (p. 99)	This is high level reporting of results with minimal methods description. Methods are reported in Nove et al. (see separate entry)
Kamuzora, P., S. Maluka, B. Ndawi, J. Byskov and A. K. Hurtig (2013). “Promoting community participation in priority setting in district health systems: experiences from Mbarali district, Tanzania.” Global Health Action 6: 22669.	“REACT was a 5-year project aimed at testing the application and effects of Accountability for Reasonableness (AFR) approach to priority setting in resource-constrained settings. AFR is a comprehensive framework which provides structure for stakeholders to establish priorities for their specific contexts, while taking into account limited resources and regulatory conditions. The REACT project aimed at implementing the four conditions of the AFR framework (see Table 3).” (p. 4)	“The aim of this article is to provide the experience of implementing community participation and the challenges of promoting it in the context of resource-poor settings, weak organizations, and fragile democratic institutions.” (p. 1)	Before and after design, with mixed qualitative data collection methods. “This article is based on two major sources of data: analysis of documents and key informant interviews. Documents analyzed included minutes of the ART, CHMT, and annual planning and priority-setting reports. Key informant interviews were conducted with various stakeholders in the district and region. Furthermore, all six representatives of the marginalized groups, namely women, youth, elderly, disabled, and people living with HIV/AIDS, who joined the CHMT for priority setting and budget discussion were interviewed. Interviews were conducted in two phases. Twenty-one interviews were carried out with various stakeholders in the district toward the end of the REACT project in August 2010. An additional 14 interviews were carried out 1 year after the end of the project in April 2012 by the researcher (S. M.) who was not directly involved in the implementation of the project in the district. In the second phase, respondents included only those who were directly involved in the priority setting and budget discussions namely CHMT members and representatives of the communities. In total, 35 interviews were carried out and analyzed” (p. 5)
Lippman, S. A., A. M. Leddy, T. B. Neilands, J. Ahern, C. MacPhail, R. G. Wagner, D. Peacock, R. Twine, D. E. Goin, F. X. Gomez-Olive, A. Selin, S. M. Tollman, K. Kahn and A. Pettifor (2018). “Village community mobilization is associated with reduced HIV incidence in young South African women participating in the HPTN 068 study cohort.” Journal of the International Aids Society 21(e25182).	Community mobilisation. "The CM intervention, conducted in partnership with Sonke Gender Justice and carried out by a trained team of mobilizers and community volunteers, sought to address intersections around HIV risk and gender norms that contribute to gender-based violence and power inequities, encouraging community members to examine how to make changes in both their own lives and in their communities through workshops and varied community activities". (p. 61) "HPTN 068 (happening simultaneously) was a randomized trial of cash transfers conditional on school attendance." (p. 61) See protocol for further details: Pettifor A, Lippman SA, Selin AM, Peacock D, Gottert A, Maman S, et al. A cluster randomized-controlled trial of a community mobilization intervention to change gender norms and reduce HIV risk in rural South Africa: study design and intervention. BMC Public Health. 2015;15:752.	“We examine the association of CM with incident HIV among AGYW (ages 13 to 21) enrolled in the HPTN 068 cohort in the Agincourt Health and socio-Demographic Surveillance System, South Africa.” (from abstract)	“Simultaneous to the HPTN 068 trial, a community mobilization programme and research initiative was underway at the Agincourt HDSS site, with implementation of a CM intervention in 11 of 22 randomly selected villages in the area. The CM intervention, conducted in partnership with Sonke Gender Justice and carried out by a trained team of mobilizers and community volunteers, sought to address intersections around HIV risk and gender norms that contribute to gender-based violence and power inequities, encouraging community members to examine how to make changes in both their own lives and in their communities through workshops and varied community activities. The intervention was evaluated using cross-sectional surveys conducted prior to (*n* = 1181) and following (*n* = 1403) the two-year intervention (2012 to 2014).” (p. 61) [process described further in protocol Pettifor et al 2015] “We examine the association of CM with incident HIV among AGYW (ages 13 to 21) enrolled in the HPTN 068 cohort in the Agincourt Health and socio-Demographic Surveillance System, South Africa. This analysis includes 2292 participants residing in 26 villages where cross-sectional, population-based surveys were conducted to measure CM among 18- to 35-year-old residents in 2012 and 2014. HPTN 068 participants completed up to five annual visits that included an HIV test (2011 to 2016). Household-level data were collected from AGYW parents/guardians and census data is updated annually. Mean village-level CM scores were created using a validated community mobilization measure with seven components (social cohesion, social control, critical consciousness, shared concerns, organizations and networks, leadership and collective action). We used pooled generalized estimating equation regression with a Poisson distribution to estimate risk ratios (RR) for the association of village-level CM score and CM components with incident HIV infection, accounting for village-level clustering and adjusting for key covariates.” (from abstract)
Miller, R. L., S. J. Reed, D. Chiaramonte, T. Strzyzykowski, H. Spring, I. D. Acevedo-Polakovich, K. Chutuape, B. Cooper-Walker, C. B. Boyer and J. M. Ellen (2017). “Structural and Community Change Outcomes of the Connect-to-Protect Coalitions: Trials and Triumphs Securing Adolescent Access to HIV Prevention, Testing, and Medical Care.” American Journal of Community Psychology 60(1-2): 199–214	“The Connect-to-Protect (C2P) Partnership for Youth Prevention Intervention was a demonstration project of the Adolescent Medicine Trials Network for HIV/AIDS Interventions (ATN) and funded by the National Institute of Child Health and Development, National Institute on Mental Health, and National Institute on Drug Abuse.” (p.5) “Coalitions included representatives of private and public health-focused organizations, organizations from other youth-focused sectors (e.g., education, juvenile justice), and prominent community institutions (e.g., businesses, churches, mayor’s offices). Coalition partners included community-based organizations specializing in youth of color, GBLTQ youth, and HIV. In many cases, youth from the target population were also members of the coalitions. Coalitions approached mobilization and planning using a structured process adapted from Fawcett et al.’s (2000) VMOSA approach. The approach was modified to incorporate root cause analysis to guide the development of a logic model linking structural and community drivers of risk and structural risk mechanisms to individual youth behaviors. Coalitions used their logic models to develop structural change objectives corresponding to the drivers and mechanisms they identified. Each objective was further delineated into action steps with target completion dates and a list of other actors needed to move the objective forward. Structural changes targeted numerous sectors in the community, including healthcare, education, criminal justice, religious, and social services.” (p. 5–6)	“...to identify the features of coalitions’ context and operation that facilitated and undermined their ability to achieve structural change and build communities’ capability to manage their local adolescent HIV epidemic effectively. In the current study, we examine the perceived contributions and accomplishments of these coalitions at the end of their lifespans to identify the features of their context and operation that facilitated and undermined their ability to achieve structural change and build capability to effectively manage their local adolescent HIV epidemic.” (pp.4–5)	“Outcome mapping”: “To identify key informants who possessed specialized knowledge of either the effects of structural changes on the systems and sectors where these effects occurred or of the cascading effects of these changes on youth, coalition staff used outcome mapping techniques. We viewed outcome mapping as an appropriate tool because of its emphasis on capturing changes in the behavior, relationships, activities, or actions of the people, groups, and organizations with whom an entity such as a coalition works. In outcome mapping, these “boundary partners” are the people through which change occurs. It is their practices and the policies they must follow in carrying out their work that coalitions are seeking to influence via structural changes. Staff nominated 293 people in 2015 and an additional 168 people in 2016 as prospective informants, for a total of 461 potential interviewees.” (p. 6) “C2P staff running the coalitions and the staff at the NCC documented coalitions’ activities, member composition, member feedback, and the status of each structural change objective on an ongoing basis and in a standardized manner. During the final 2 years of the project (2014–2016), 318 key informant interviews with youth and community leaders were conducted by an external evaluation team based at Michigan State University” (p. 6) “Coalition leaders may have disproportionately recommended informants who possessed positively biased views of the coalition and a small number of informants who were likely to offer critical views on coalition accomplishments or functioning. […] We sought to guard against this possibility by asking for detailed descriptions of changes and actions, with a focus on observed changes in the 2 years prior to each interview. We asked for evidence in support of every claim of positive impact. We limited our analysis to changes that corresponded with accomplished objectives from the coalition’s and NCC’s records, as these could be most clearly attributed to the coalition’s work. Nonetheless, the sample of informants may have provided us with an unduly favorable view of the coalitions’ accomplishments, painting a picture of them as more successful and impactful than warranted.” (p. 18)
Nove, A., L. Hulton, A. Martin-Hilber and Z. Matthews (2014). “Establishing a baseline to measure change in political will and the use of data for decision-making in maternal and newborn health in six African countries.” Int J Gynaecol Obstet 127(1): 102-107.	E4A (see Hulton et al) “The Evidence for Action (E4A) program assumes that both resource allocation and quality of care can improve via a strategy that combines evidence and advocacy to stimulate accountability.” (from abstract)	“The questions for E4A therefore were: how could political will be measured; to what extent did decision-makers have access to and use data; and how could change over time in these two key outcomes be measured? To help answer them and determine the baseline situation for the program, we designed two tools: the Politics, Power, and Perceptions (PPP) tool and the Data for Decision-Making (DDM) tool” (p. 102)	Note: the authors report the ‘independent’ study was designed before the country teams had been recruited, limiting the ownership of the study by the country teams “and consequently much time and effort was required to explain the value of the data to them, and to encourage them to use the data to help plan their strategy” (p. 103) Design: Repeat cross-sectional survey with repeat interviews with respondents in each phase where possible. Sample: Purposive sampling “In each country, independent consultants were contracted to select and interview a purposive sample of 40–60 key informants for each tool, to gather views from an appropriate spread of national level, district level, and facility level informants. At national and district level (here district refers to the subnational level that was appropriate for each country), the pool of eligible informants was relatively small and the aim was to interview as many as possible. At facility level, the sampling was done by listing all possible health facilities in the E4A focal areas, then selecting a subsample based on how practical it was to visit them within the allotted time. At each sampled facility, contractors were instructed to interview 1–3 eligible informants according to the informants’ availability on the day of the visit.” (p. 103) Data collection methods: two questionnaires. “The PPP tool assesses the level of political will to improve MNH outcomes. The DDM tool assesses the extent to which key stakeholders make use of MNH data. The use of face-to-face interviews allowed for a detailed set of questions (average interview duration was 20 min for PPP and 30 min for DDM) and for interviewers to request documentary evidence to back up the responses given by DDM informants, which acted as an important quality control mechanism. However, the use of a structured questionnaire meant informants’ answers could not be explored in more detail to gain more qualitative insight.” (p. 103)
Samuel, J. (2016). “The role of civil society in strengthening intercultural maternal health care in local health facilities: Puno, Peru.” Global Health Action 9(1)1.	“The initiative recruited, trained, and supported Quechua speaking indigenous women from community-based organizations (CBOs) in the department of Puno to act as volunteer citizen monitors to observe and report on the delivery of health care services in their local publicly provided facilities. Lawyers from the Puno office of the Defensor$\overset{\overset{\acute{\mkern6mu}}{\kern0.333em }}{\mathrm{i}}$a del Pueblo, Peru’s National Human Rights Ombudsman’s Office, also provided the monitors with training and support, as did other strategic allies.” (p. 2)	This article examines whether a grassroots accountability initiative based on citizen monitoring of local health facilities by indigenous women can help to promote the objectives of the intercultural birthing policy and improve intercultural maternal health care.	“The findings presented here are drawn from a larger qualitative research study that included fieldwork conducted in 2010 and 2011. Methodologically, this study used an institutional ethnographic approach to examine the work of citizen monitors in Puno, Peru. Institutional ethnography is based on the premise that analyzing the work processes and other experiences of a particular group of people can provide an important vantage point to understand a broader set of social and institutional relations. The author uses the notions of work processes and work knowledge to help explore and understand the work, roles, and working relationships of the citizen monitors in Puno. This involves an in-depth examination of the daily monitoring work done by this group of women to promote change in reproductive health service delivery. This approach is well suited to gain insight into the complex power relations that shape the monitors’ unequal engagement with their local health facilities.” (p. 3)
Schaaf, M., S. M. Topp and M. Ngulube (2017). “From favours to entitlements: community voice and action and health service quality in Zambia.” Health Policy Plan 32(6): 847–859.	“The primary objective of Citizen Voice and Action (CVA) is to increase dialogue and accountability between three groups: citizens, public service providers and government officials (political and administration) to improve the delivery of public services” (p.849) “The program occurs in three, iterative phases. The first phase entails World Vision (WV)-led relationship building with communities and service providers and stakeholder mobilization to inform the community and relevant actors about the goals and components of CVA. Next, WV convenes an open community gathering during which a CVA Committee is formed, usually by a consensus process. About 10–15 people join; membership is voluntary. CVA Committee members are often also members of other community structures, such as village development committees and neighborhood health committees. Insofar as possible, WV tries to facilitate the creation of a diverse CVA Committee, so that the Committee has widespread legitimacy. Following facilitation from WV, representatives from the government educate communities about relevant legislation and national service delivery standards. Citizens may have preferences and priorities that are not formally enshrined in national standards, thus they also articulate standards (“perception-based indicators”) that they think their local facility should meet. In the second phase, the health facility’s (or other service provider’s, depending on the context) realization of both perception-based indicators and national service delivery standards are assessed. A social audit process is used with service providers and communities to assess performance of the clinics against national service delivery standards. Here, citizens and service providers observe the facility and look at facility data to assess to what extent the facility is compliant with national service delivery standards. Then, citizens and service providers use community score cards to rate their health facilities against the perception-based indicators. Third, citizens, local elected representatives and service providers, convene interface meetings. They discuss the service delivery gaps identified and elaborate action plans to address some of these challenges. Action plans identify individuals and groups responsible for each action. The plans are then implemented and monitored in subsequent interface meetings. The three phases are repeated, as communities and the government tackle increasingly difficult challenges.” (p. 849)	“We sought to make tentative, contextualized programmatic and theoretical propositions about how the CVA program theory was realized in the health sector in 3 of Zambia’s 103 districts. The study aimed to answer: 1. How does CVA affect the relationship between citizens and the health sector? 2. How does the health sector respond to CVA? 3. What elements of context facilitate or hinder positive change in the health sector in response to CVA?” (p. 850)	“A full-fledged realist evaluation would typically require longitudinal engagement with program participants and stakeholders. Moreover, given CVA’s widespread use, a rigorous realist evaluation would entail looking at multiple countries. Thus, we describe this study as a realist informed qualitative study, an approach that has been taken in other contexts where researchers feel that the context, mechanisms, and outcomes framing would add value to extant data” (p. 850) Data collection methods: “Secondary data were used iteratively. Secondary data included WV program documents, score cards and action plans generated by CVA activities, and materials WV developed summarizing health entitlements. More importantly, we also reviewed articles regarding social accountability in all domains (not just health), as well as health systems and policy research articles relating to relationships within health systems and between communities and the health system.” (p. 851). “Primary data were collected between November 2013 and January 2014. CVA had started in these communities in 2008. At the time the research was conducted, the program was ongoing in all of them. Methods used included in-depth interviews with district health officials (*n* = 5), traditional community leaders (*n* = 2), rural health center staff from one facility in each of the three sites (*n* = 4), WV staff based in the districts under study (*n*¼8), and WV staff based in Lusaka (*n* = 1). Focus groups were also conducted with CVA members in each of the three sites (*n* = 27).” (p. 851)
Sebert Kuhlmann, A. K., S. Gullo, C. Galavotti, C. Grant, M. Cavatore and S. Posnock (2017). “Women's and Health Workers’ Voices in Open, Inclusive Communities and Effective Spaces (VOICES): Measuring Governance Outcomes in Reproductive and Maternal Health Programmes.” Development Policy Review 35(2): 289–311.	CARE Community Score Card (CSC): “The theory of change for the CSC is based on CARE International’s Governance Programming Framework (GPF). The adaptation of the GPF to maternal and reproductive health focuses on three key ingredients: 1) empowering women and service users, 2) empowering health care service providers, and 3) creating spaces for service users and service providers to engage in constructive dialogue and negotiation. “ (p. 291)	“A cluster-randomized trial of the CSC is being conducted in Malawi, focused on reproductive and maternal health service delivery and outcomes. As part of the baseline data collection for the randomized trial, measures of key components of the theory of change were developed. These measures are part of two multidimensional survey tools—one for women called Women’s Voices in Open, Inclusive Communities and Effective Spaces (VOICES) and another for health workers called Health Worker’s VOICES. These same survey tools, with some minor modifications resulting from the analyses presented here, will be used during the end line data collection for the randomized trial in order to assess change over time as a result of the CSC intervention.” (p. 292)	“We examined the psychometric properties of the measures in each of these domains using baseline data from the cluster-randomized trial in Malawi. Data presented here were collected in Ntcheu district, Central Region, Malawi from October to December 2012. The evaluation uses a cluster-randomized control design with ten matched pairs of health facilities and surrounding catchment areas. Matching criteria included presence (or absence) of basic emergency obstetric services, facility administrator (MOH or CHAM), proximity to the Mozambique border and population size of the catchment area. One of each pair was randomly assigned to the intervention arm. Two cross-sectional surveys, one of women and one of health workers were conducted at baseline. In the 20 catchment areas, Women’s VOICES surveyed women aged from 15 to 49 who had given birth within the past 12 months—regardless of whether they had delivered in a health facility or not—and whose babies were still living, using a two stage probability proportional to size (PPS) methodology […] All 327 health workers (both facility- and community-based) within the 20 catchment areas were eligible for the Health Workers’ VOICES survey.” (p. 295)
Topp, S. M., J. Black, M. Morrow, J. M. Chipukuma and W. Van Damme (2015). “The impact of human immunodeficiency virus (HIV) service scale-up on mechanisms of accountability in Zambian primary health centres: a case-based health systems analysis.” BMC Health Services Research 15: 67.	Paper describes scale up of HIV services, and looks at social accountability as part of that. The main mechanism for social accountability was Neighborhood Health Committees (NHCs)	“The explicit focus of this article is to examine whether and how the establishment and scale-up of HIV services influenced mechanisms of accountability within the primary service domain, and, as a result, service quality and responsiveness. We then apply these findings to a consideration of whether there is merit in attempting to design disease specific interventions that reflect the complexity in primary level services, and, in the process, enable a more contextually comprehensive approach to the design and implementation of health system strengthening interventions.” (p. 2)	“We adopted a multi-case study design using a theoretical replication strategy. Case ‘units’—four primary health centres located in two adjacent Districts, one urban one rural—were selected by the lead investigator (SMT) in consultation with District Medical Officers, and based on both empiric and anecdotal evidence of characteristics that enabled exploration of patterns of service delivery. Such characteristics included: average patient attendance data; vaccination coverage rates; and District officers’ descriptions of health center performance. […]Methods used at each case site included: in-depth interviews with a proportionate sample of healthcare workers from various levels (*n* = 60); semi-structured interviews with a quasi-random sample of patients (*n* = 180); review of health center paperbased registers; and direct observation of facility operations […] In addition, key informant interviews were held with government and non-government officials (*n* = 14) with specific knowledge or experience of the processes of HIV service scale-up.” (pp. 4–5)

Qualitative data were essential to provide information about accountability mechanisms, and to support causal claims that were sometimes only weakly supported by the quantitative data alone. For example, this was the case in the many studies where the quantitative data were before-and-after type data that could have been biased by secular trends, i.e., where it would be difficult to make credible causal claims based only on those data. Qualitative data were primarily generated via interviews, focus group discussions, and ethnographic methods including observations.

Additionally, some papers contained broader structural analysis contextualizing interventions in relation to relevant, longstanding processes of marginalization. For instance, Dasgupta 2011 notes that “in addition to the health system issues discussed [earlier in the paper], the duty bearers appear to hold a world view that precludes seeing Dalit and other disadvantaged women as human beings of equivalent worth: you can in fact die even after reaching a well-resourced institution if you are likely to be turned away or harassed for money and denied care” [18, p. 9].

There were very few outcome measures reported in the studies which directly related to social accountability. Instead, they usually related to the intervention (e.g., number of meetings, number of action points recorded). Outcome measures included quantitative process measures such as total participants attending meetings (e.g., [16]), how many calls were made to a hotline (e.g., [24]), numbers of services provided, and outcome measures such as measures of satisfaction (e.g., [25, 32, 33]). Qualitative studies examined how changes had been achieved (for instance by exploring involvement of civil society organisations in promotion and advocacy), or perceptions of programme improvement (e.g., [20, 22, 34]). Many of the health outcomes were reported using proxy measures (e.g., home visits from a community health worker, care-seeking) [32, 35].

There were various attempts to capture the impact of the intervention on decision-making and policy change. For example, “process tracing” was used, “to assess whether and how scorecard process contributed to changes in policies or changes in attitudes or practices among key stakeholders” [23 p. 374], and “outcome mapping” (defined as, “emphasis on capturing changes in the behavior, relationships, activities, or actions of the people, groups, and organizations with whom an entity such as a coalition works”) [27, p. 6] was used to assess effects of the intervention on systems and staff.

### Theoretical frameworks

In 10 out of 22 cases, we found an explicit theoretical framework that guided the evaluation of the intervention. In some additional cases, there appeared to be an implicit theoretical approach or there is reference to a “theory of change” but these were not spelled out clearly.

### Harms or negative effects reported

Studies which emphasised quantitative data either alone or as a part of a mixed methods data collection strategy did not report harms or intent to measure any. The only studies reporting negative aspects of the intervention—either its implementation or its effects—emphasised qualitative data in their reporting. Not all qualitative studies reported negative aspects of the intervention, but it was notable that the more detailed qualitative work considered a wider range of possible outcomes including unintended or undesirable outcomes.

Studies reporting any types of negative effects varied in terms of the type of harms or other negative aspects of interventions reported, although complex relationships with donors was mentioned more than once. For instance, Aveling et al note:

…relations of dependence encourage accountability toward donors, rather than to the communities which interventions aim to serve […] far more time is spent clarifying reporting procedures and discussing strategies to meet high quantitative targets than is spent discussing how to develop peer facilitators’ skills or strategies to facilitate participatory peer education. [22, p. 1594–5]

Some authors did not report on negative effects as such, but did acknowledge the limitations of the interventions they examined—for instance, that encouraging communities to speak out about problems will not necessarily be enough to promote improvement [16]. Similarly Dasgupta reported how, “[t]he unrelenting media coverage of corruption in hospitals, maternal and infant deaths and the dysfunctional aspects of the health system over the last six years, occasionally spurred the health department to take some action, though usually against the lowest cadre of staff” [18 p. 7] and “[w]hen civil society organizations, speaking on behalf of the poor initially mediated the rights-claiming to address powerful policy actors such as the Chief Minister, it did not stimulate any accountability mechanism within the state to address the issue” [18p. 7]. In their 2015 study, Dasgupta et al. address the potential harms that could have been caused by the intervention—a hotline for individuals to report demands for informal payments—and explain how the intervention was designed to avoid these [24].

### Costs and sustainability

Only four studies contained even passing reference to the cost or sustainability of the interventions. One study indicated that reproductive health services had been secured for soldiers and their wives [22]. One mentioned that although direct assistance had ceased, activities continued with technical support provided on a volunteer basis [28], one (a protocol) set out how costs would be calculated in the final study [26], and one mentioned in passing that a district had not allocated funds to cover costs associated with additional stakeholders [20].

Challenges to sustainability were noted in several studies [16, 20–25, 32, 33].

### Accountability of the authors to the reader

Very few studies specified the relationship between the evaluation team and the implementation team and in many cases, they appear to be the same team, or have team members in common. In most cases, there was no clear statement explaining any relationships that might be considered to constitute a conflict of interest, or how these were handled.

Information about evaluation funding was more often provided, although again it was not clear whether the funder had also funded the intervention, or if they had, to what extent the evaluation was conducted independently from the funders.

## Discussion

Most studies reported a mix of qualitative and quantitative data, with most analyses based on the qualitative data. Two studies used a trial design to test the intervention—one examined the effects of implementing CARE community score cards [32] and the other tested the effects of a community mobilization intervention [36]. This relative lack of trials is notable given the number of trials related to social accountability in other sectors [3, 9]. The more exploratory studies which attempted to capture aspects of the interventions—such as how they were taken up—used predominantly qualitative data collection methods.

The studies we identified show the clear benefits of including qualitative data collection to assess social accountability processes and outcomes, with indicative quantitative data to assess specific health or service improvement outcomes. High-quality collection and analysis of qualitative data should be considered as at least a part of subsequent studies in this complex area. The “pure” qualitative studies were the only ones where any less-positive findings about the interventions were reported, perhaps because of the emphasis on reflexivity in analysis of qualitative data, which might encourage transparency. We were curious about whether there was any relationship between harms being reported and independence of studies from the funded intervention, but we found no particular evidence from our included studies to indicate any association. One study mentioned that lack of in-country participation in the design process led to lack of interest in using the findings to help plan country strategy [31].

It was notable that studies often did not specify their evaluation methods clearly. In these cases, methods sections of the papers were devoted to discussing methods for the intervention rather than its evaluation.

When trying to measure interventions intended to influence complex systems (as social accountability interventions attempt to do), it is important to understand what the intervention intends to change and why in order to assess whether its effects are as expected, and understand how any effects have been achieved. There was a notable lack of any such specification in many of the included studies. For example, there were few theoretical frameworks cited to support choices made about evaluation methods and, related to this, there were few references to relevant literature that might have informed both the interventions and the evaluation methodologies. The literature on public and patient involvement, for instance, was not mentioned despite this literature containing relevant experiences of trying to evaluate these types of complex, participatory processes in health. It is possible that some of the studies were guided by hypotheses and theoretical frameworks that were not described in the papers we retrieved.

Sustainability of the interventions and their effects after the funded period of the intervention was rarely discussed or examined. A small, enduring change for the better that also creates positive ripple effects over time may be preferable to larger, temporary effects that end with the end of the intervention funding. It would also be useful to discuss with funders and communities in advance what type of outcome would indicate success and over what period of time, to ensure that measures take into account what is considered important to the people who will use them. Sustainability and effectiveness are known to diminish after the funded period of the intervention [37]. Longer term follow-up may be hindered because of the way funding is generally allocated over short periods. It would be interesting to see a greater number of longer-term follow up studies examining what happened “after” the intervention had finished in order to inform policymakers about what the truly “cost-effective” programmes are likely to be. For example, some studies have traced unfolding outcomes after the intervention has finished; these may be important to take into account in any effectiveness considerations.

There was little transparency about funding and any conflicts of interest—which seemed surprising in studies of social accountability interventions. We strongly recommend that these details be provided in future work and be required by journals before publication.

A limitation of this study was that our searches yielded studies where accountability of health workers to communities or to donors appeared to be the main area of interest. A broader understanding of accountability might yield further useful insights. For instance, it seems likely that an intersectional perspective might put different forms of social accountability in the spotlight (e.g., retribution or justice connected with sexual violence or war crime, examining the differentiated effects on sexual and reproductive health, rather than solely accountability in a more bounded sense) [38]. By limiting our view of what “accountability” interventions can address within health, we may unintentionally imply broader questions of accountability are not relevant—e.g., effects of accountability in policing practices on health, effects of accountability in education policy on health, and so on.

With only a few notable exceptions, we lack broader sociohistorical accounts of the ways in which these interventions are influenced by the political, historical, and geographical context in which they appear, and how dynamic social change and “tipping point” events might interrelate with the official “intervention” activities—pushing the intervention on, or holding it back, co-opting it for political ends, or losing control of it completely during civil unrest. While the studies we identified did use more qualitative approaches to assessing what had happened during interventions, the scope of the studies was often far narrower than this—for instance lacking information on broader political issues that affected the intervention at different points in time. In future, studies examining health effects of social accountability interventions should consider taking a more theoretical approach—setting out in more detail what social processes are happening in what historical/geographical/social context so that studies develop a deeper understanding, including using and further developing theories of social change to improve the transferability of the findings. For instance, lessons on conducting and evaluating patient involvement interventions in the UK may well have a bearing on improving social accountability and its measurement in India and vice versa. Related to this, we note that although there is clear guidance from the evaluation literature that it is important to take a systems approach to understanding complex interventions, none of our included studies explicitly took a systems approach—applying these types of approaches more systematically to social accountability interventions is a fertile area for future investigation. Without such studies, we risk implying that frontline workers are the only site of “accountability” and, by omission, fail to examine the role of more powerful actors and social structures which may act to limit the options of frontline workers, as well as failing to explore and address the ways in which existing structural inequalities might hamper equitable provision and uptake of health services.

Terminology may be hampering transfer of theoretically relevant material into and out of the “social accountability” field. The term “social accountability” may imply an adversarial relationship where certain individuals are acting in bad faith. One of the studies in our review used different terminology—“collaborative synergy”—referring to the work of coalitions in the Connect2Protect intervention [27]. We speculate that lack of agreed, common terminology may hinder learning from other areas of research—the phrase “social accountability” is not commonly used in the patient and public involvement (PPI) literature, possibly because of the greater emphasis in high income settings on co-production and sustainability compared with more of a “policing” emphasis in the literature reporting on LMIC settings. Yet one of the purposes of PPI interventions is to improve services and this may well include healthcare providers being held accountable for the services they provide. Litigation was outside the scope of this article, but legally enshrined rights to better healthcare are crucial and litigation is a key route to ensuring these rights are achieved in practice. A more nuanced account of these types of interventions in context would be valuable in understanding “what works where and why,” to inform future policy and programmes.

Dasgupta et al. comment on how hard it is to attribute change to any particular aspect of a social accountability intervention because successful efforts are led by individuals in many different roles whose relationships with one another are constantly changing and adapting. Attributing success is difficult because these changing relationships shape how and whether any individual can have an impact through their actions.

Evaluation tools, particularly those used within and for a specific time frame, have a limited capacity to capture the iterative nature of social accountability campaigns, as well as to measure important impacts like empowerment, changes in the structures that give rise to rights violations, and changes in relationships between the government and citizens. [24, p. 140]

## Conclusions

Designing adequate evaluation strategies for social accountability interventions is challenging. It can be difficult to define the boundaries of the intervention (e.g., to what extent does it make conceptual sense to report on the intervention without detailing the very specific social context?), or the boundaries of what should be evaluated (e.g., political change or only changes in specific health outcomes). What is clear is that quantitative measures are generally too limited on their own to provide useful data on attribution, and the majority of evaluations appear to acknowledge this by including qualitative data as part of the evidence. The goals and processes of the interventions are inherently social. By examining social dimensions in detail, studies can start to provide useful information about what could work elsewhere, or provide ideas that others can adapt to their settings. More lessons should be drawn from existing evaluation and accountability work in high-income settings—the apparent lack of cross-learning or collaborative working between HIC and LMIC settings is a wasted opportunity, particularly when so much good practice exists in HIC and in LMIC settings—there are ample opportunities to learn from one another that are often not taken up and this is clear from the literature which tends to be siloed along country-income lines. Finally, more transparency about funding and histories of these interventions is essential.

## Data Availability

Not applicable.

## Footnotes

1 https://www.crd.york.ac.uk/prospero/display_record.php?RecordID=108252
